# Supplementary material for: Network Biology-Inspired Machine Learning Features Predict Cancer Gene Targets and Reveal Target Coordinating Mechanisms
Source: Pharmaceuticals (Basel). 2023 May 16;16(5):752. doi: 10.3390/ph16050752 (PMC10223789; doi:10.3390/ph16050752)
Supplement: Supplementary file 1 [file pharmaceuticals-16-00752-s001.zip › pharmaceuticals-2352850-supplementary.pdf]

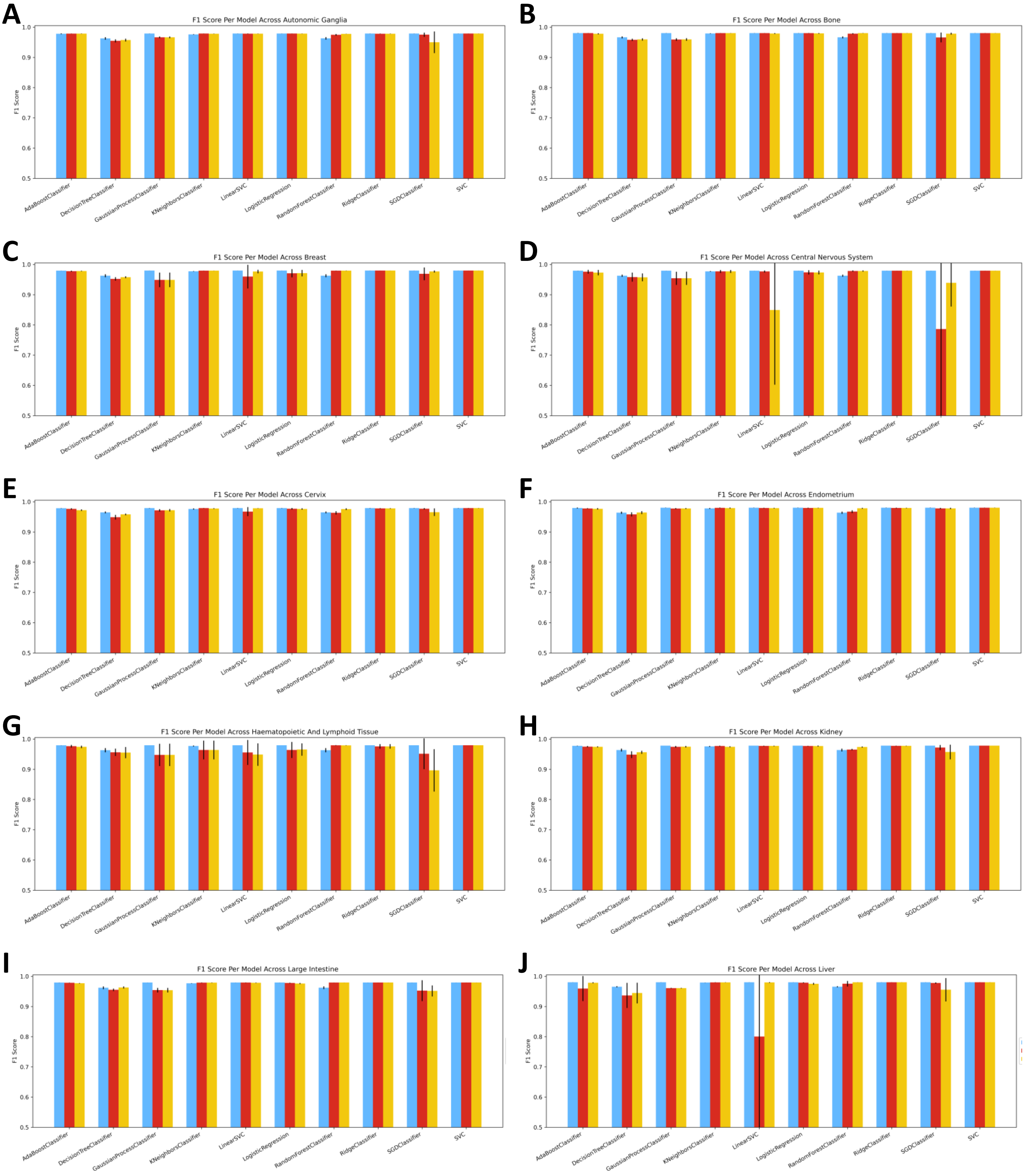
A
F1 Score Per Model Across Autonomic Ganglia
B
F1 Score Per Model Across Bone
C
F1 Score Per Model Across Breast
D
F1 Score Per Model Across Central Nervous System
E
F1 Score Per Model Across Cervix
F
F1 Score Per Model Across Endometrium
G
F1 Score Per Model Across Haematopoietic And Lymphoid Tissue
H
F1 Score Per Model Across Kidney
I
F1 Score Per Model Across Large Intestine
J
F1 Score Per Model Across Liver
F1 Score
1.0
0.9
0.8
0.7
0.6
0.5
AdaBoostClassifier
DecisionTreeClassifier
GaussianProcessClassifier
KNeighborsClassifier
LinearSVC
LogisticRegression
RandomForestClassifier
RidgeClassifier
SGDClassifier
SVC

Figure S1

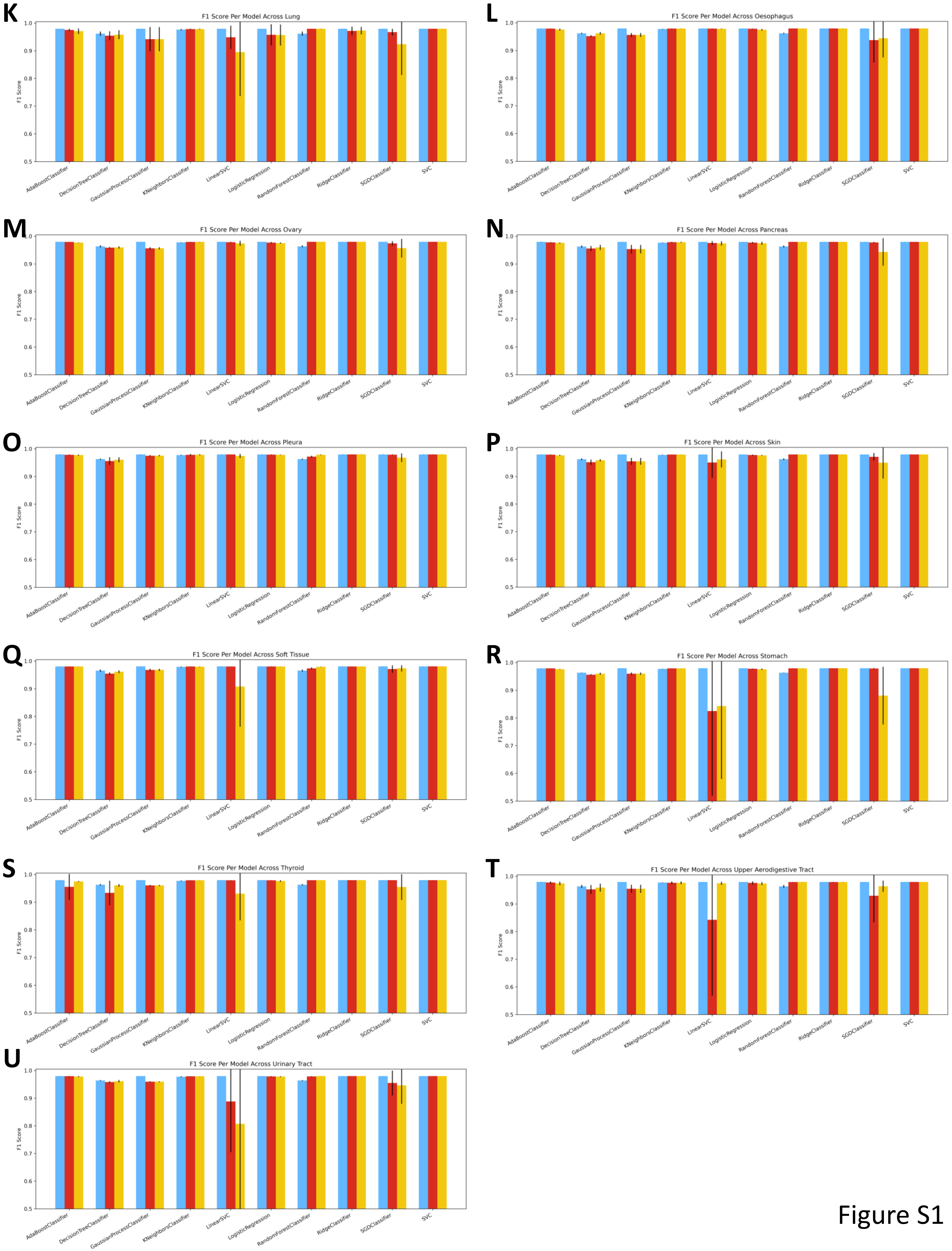
K
F1 Score Per Model Across Lung
L
F1 Score Per Model Across Oesophagus
M
F1 Score Per Model Across Ovary
N
F1 Score Per Model Across Pancreas
O
F1 Score Per Model Across Pleura
P
F1 Score Per Model Across Skin
Q
F1 Score Per Model Across Soft Tissue
R
F1 Score Per Model Across Stomach
S
F1 Score Per Model Across Thyroid
T
F1 Score Per Model Across Upper Aerodigestive Tract
U
F1 Score Per Model Across Urinary Tract
F1 Score
1.0
0.9
0.8
0.7
0.6
0.5
AdaBoostClassifier
DecisionTreeClassifier
GaussianProcessClassifier
KNeighborsClassifier
LinearSVC
LogisticRegression
RandomForestClassifier
RidgeClassifier
SGDClassifier
SVC

Figure S1

A

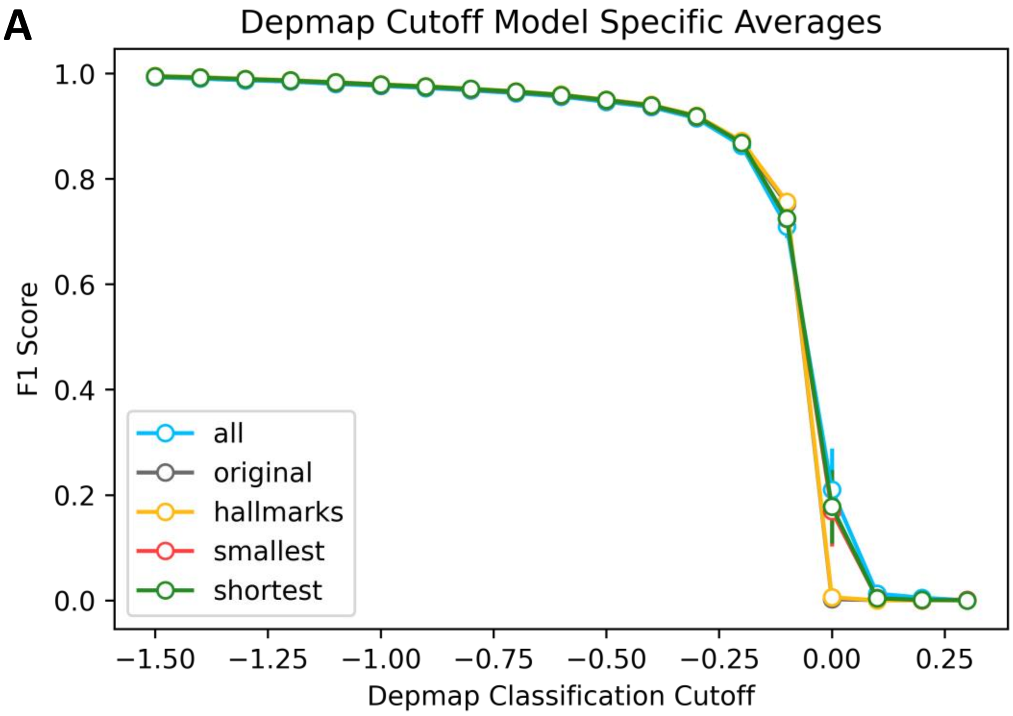
Depmap Cutoff Model Specific Averages
1.0
0.8
0.6
0.4
0.2
0.0
F1 Score
all
original
hallmarks
smallest
shortest
−1.50
−1.25
−1.00
−0.75
−0.50
−0.25
0.00
0.25
Depmap Classification Cutoff

Figure S2

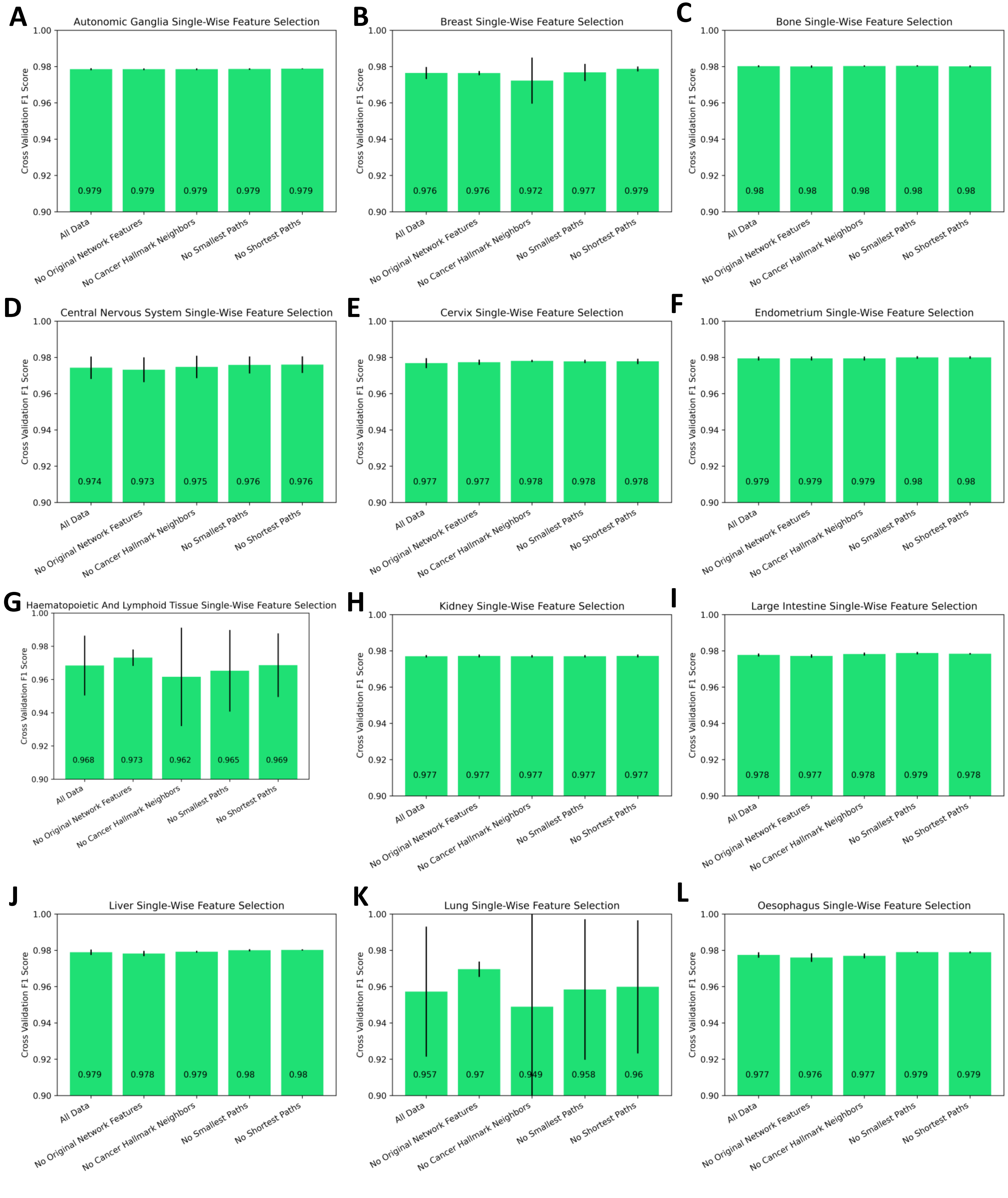
A
Autonomic Ganglia Single-Wise Feature Selection
0.979 0.979 0.979 0.979 0.979
B
Breast Single-Wise Feature Selection
0.976 0.976 0.972 0.977 0.979
C
Bone Single-Wise Feature Selection
0.98 0.98 0.98 0.98 0.98
D
Central Nervous System Single-Wise Feature Selection
0.974 0.973 0.975 0.976 0.976
E
Cervix Single-Wise Feature Selection
0.977 0.977 0.978 0.978 0.978
F
Endometrium Single-Wise Feature Selection
0.979 0.979 0.979 0.98 0.98
G
Haematopoietic And Lymphoid Tissue Single-Wise Feature Selection
0.968 0.973 0.962 0.965 0.969
H
Kidney Single-Wise Feature Selection
0.977 0.977 0.977 0.977 0.977
I
Large Intestine Single-Wise Feature Selection
0.978 0.977 0.978 0.979 0.978
J
Liver Single-Wise Feature Selection
0.979 0.978 0.979 0.98 0.98
K
Lung Single-Wise Feature Selection
0.957 0.97 0.949 0.958 0.96
L
Oesophagus Single-Wise Feature Selection
0.977 0.976 0.977 0.979 0.979
Cross Validation F1 Score
0.90 0.92 0.94 0.96 0.98 1.00
All Data
No Original Network Features
No Cancer Hallmark Neighbors
No Smallest Paths
No Shortest Paths

Figure S3

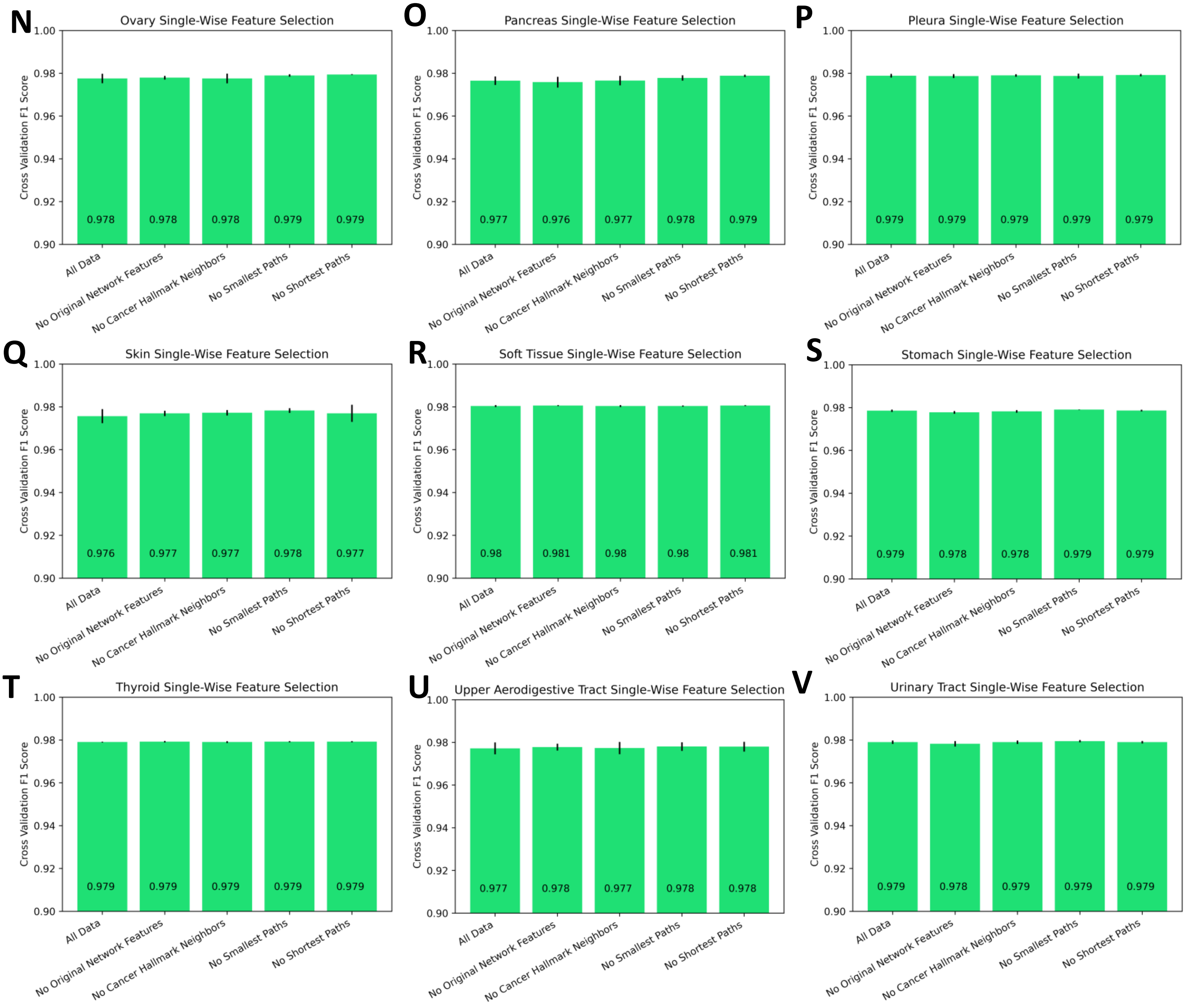
N
Ovary Single-Wise Feature Selection
Cross Validation F1 Score
1.00
0.98
0.96
0.94
0.92
0.90
0.978
0.978
0.978
0.979
0.979
All Data
No Original Network Features
No Cancer Hallmark Neighbors
No Smallest Paths
No Shortest Paths
O
Pancreas Single-Wise Feature Selection
0.977
0.976
0.977
0.978
0.979
P
Pleura Single-Wise Feature Selection
0.979
0.979
0.979
0.979
0.979
Q
Skin Single-Wise Feature Selection
0.976
0.977
0.977
0.978
0.977
R
Soft Tissue Single-Wise Feature Selection
0.98
0.981
0.98
0.98
0.981
S
Stomach Single-Wise Feature Selection
0.979
0.978
0.978
0.979
0.979
T
Thyroid Single-Wise Feature Selection
0.979
0.979
0.979
0.979
0.979
U
Upper Aerodigestive Tract Single-Wise Feature Selection
0.977
0.978
0.977
0.978
0.978
V
Urinary Tract Single-Wise Feature Selection
0.979
0.978
0.979
0.979
0.979

Figure S3

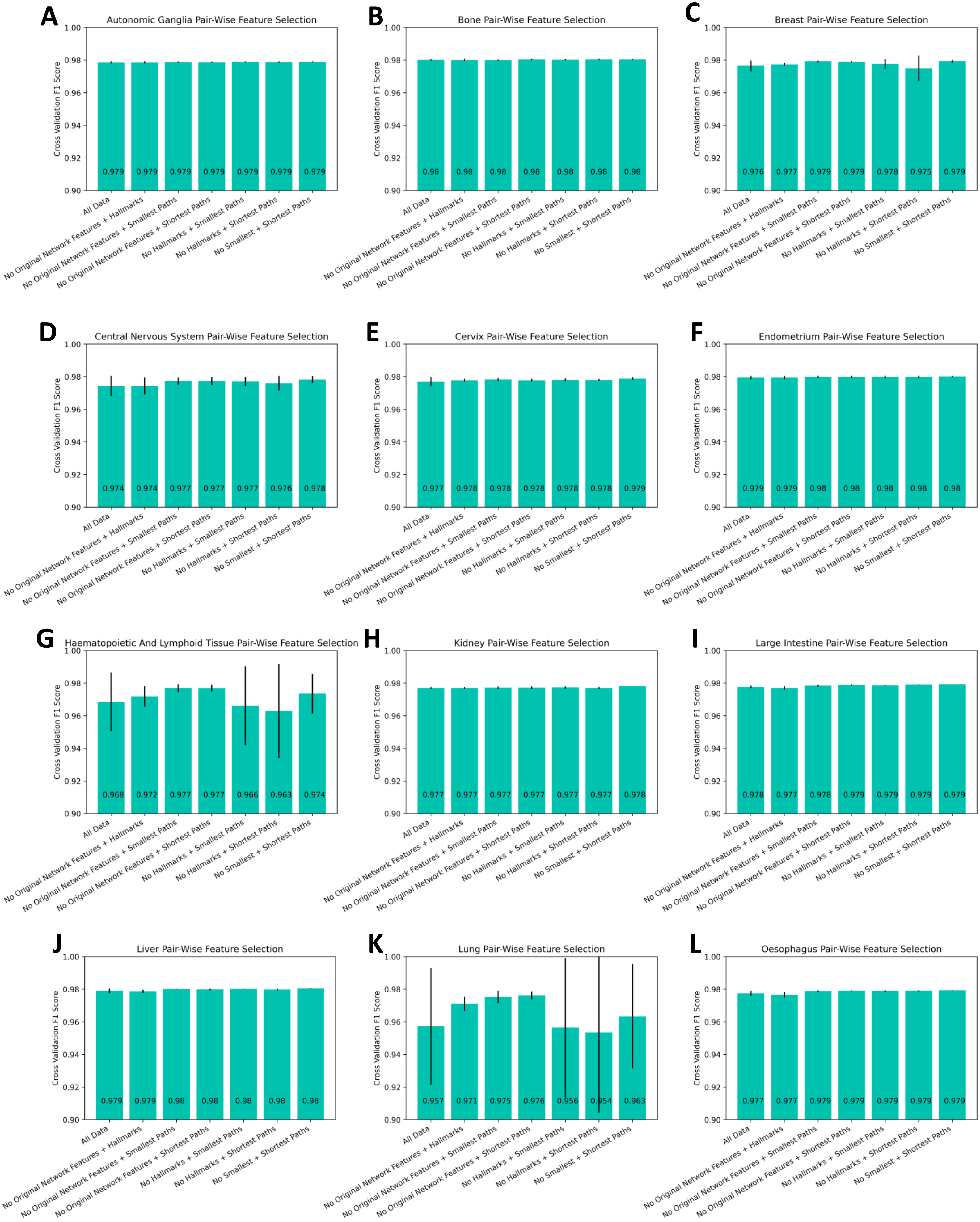
A
Autonomic Ganglia Pair-Wise Feature Selection
B
Bone Pair-Wise Feature Selection
C
Breast Pair-Wise Feature Selection
D
Central Nervous System Pair-Wise Feature Selection
E
Cervix Pair-Wise Feature Selection
F
Endometrium Pair-Wise Feature Selection
G
Haematopoietic And Lymphoid Tissue Pair-Wise Feature Selection
H
Kidney Pair-Wise Feature Selection
I
Large Intestine Pair-Wise Feature Selection
J
Liver Pair-Wise Feature Selection
K
Lung Pair-Wise Feature Selection
L
Oesophagus Pair-Wise Feature Selection
Cross Validation F1 Score
1.00
0.98
0.96
0.94
0.92
0.90
All Data
No Original Network Features + Hallmarks
No Original Network Features + Smallest Paths
No Original Network Features + Shortest Paths
No Hallmarks + Smallest Paths
No Hallmarks + Shortest Paths
No Smallest + Shortest Paths
A: 0.979 0.979 0.979 0.979 0.979 0.979 0.979
B: 0.98 0.98 0.98 0.98 0.98 0.98 0.98
C: 0.976 0.977 0.979 0.979 0.978 0.975 0.979
D: 0.974 0.974 0.977 0.977 0.977 0.976 0.978
E: 0.977 0.978 0.978 0.978 0.978 0.978 0.979
F: 0.979 0.979 0.98 0.98 0.98 0.98 0.98
G: 0.968 0.972 0.977 0.977 0.966 0.963 0.974
H: 0.977 0.977 0.977 0.977 0.977 0.977 0.978
I: 0.978 0.977 0.978 0.979 0.979 0.979 0.979
J: 0.979 0.979 0.98 0.98 0.98 0.98 0.98
K: 0.957 0.971 0.975 0.976 0.956 0.954 0.963
L: 0.977 0.977 0.979 0.979 0.979 0.979 0.979

Figure S4

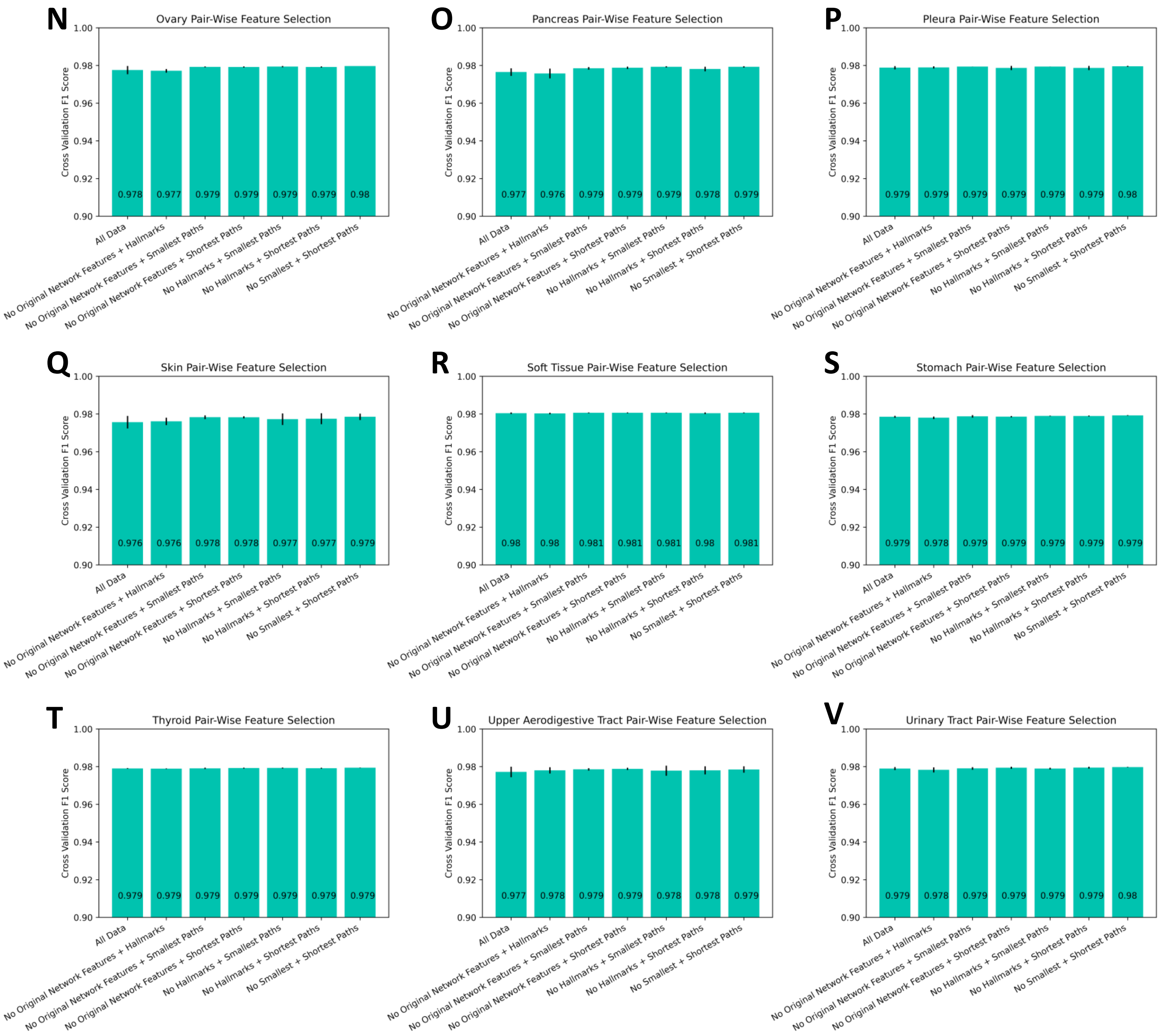
N
Ovary Pair-Wise Feature Selection
O
Pancreas Pair-Wise Feature Selection
P
Pleura Pair-Wise Feature Selection
Q
Skin Pair-Wise Feature Selection
R
Soft Tissue Pair-Wise Feature Selection
S
Stomach Pair-Wise Feature Selection
T
Thyroid Pair-Wise Feature Selection
U
Upper Aerodigestive Tract Pair-Wise Feature Selection
V
Urinary Tract Pair-Wise Feature Selection
Cross Validation F1 Score
1.00
0.98
0.96
0.94
0.92
0.90
All Data
No Original Network Features + Hallmarks
No Original Network Features + Smallest Paths
No Original Network Features + Shortest Paths
No Hallmarks + Smallest Paths
No Hallmarks + Shortest Paths
No Smallest + Shortest Paths
0.978 0.977 0.979 0.979 0.979 0.979 0.98
0.977 0.976 0.979 0.979 0.979 0.978 0.979
0.979 0.979 0.979 0.979 0.979 0.979 0.98
0.976 0.976 0.978 0.978 0.977 0.977 0.979
0.98 0.98 0.981 0.981 0.981 0.98 0.981
0.979 0.978 0.979 0.979 0.979 0.979 0.979
0.979 0.979 0.979 0.979 0.979 0.979 0.979
0.977 0.978 0.979 0.979 0.978 0.978 0.979
0.979 0.978 0.979 0.979 0.979 0.979 0.98

Figure S4

A

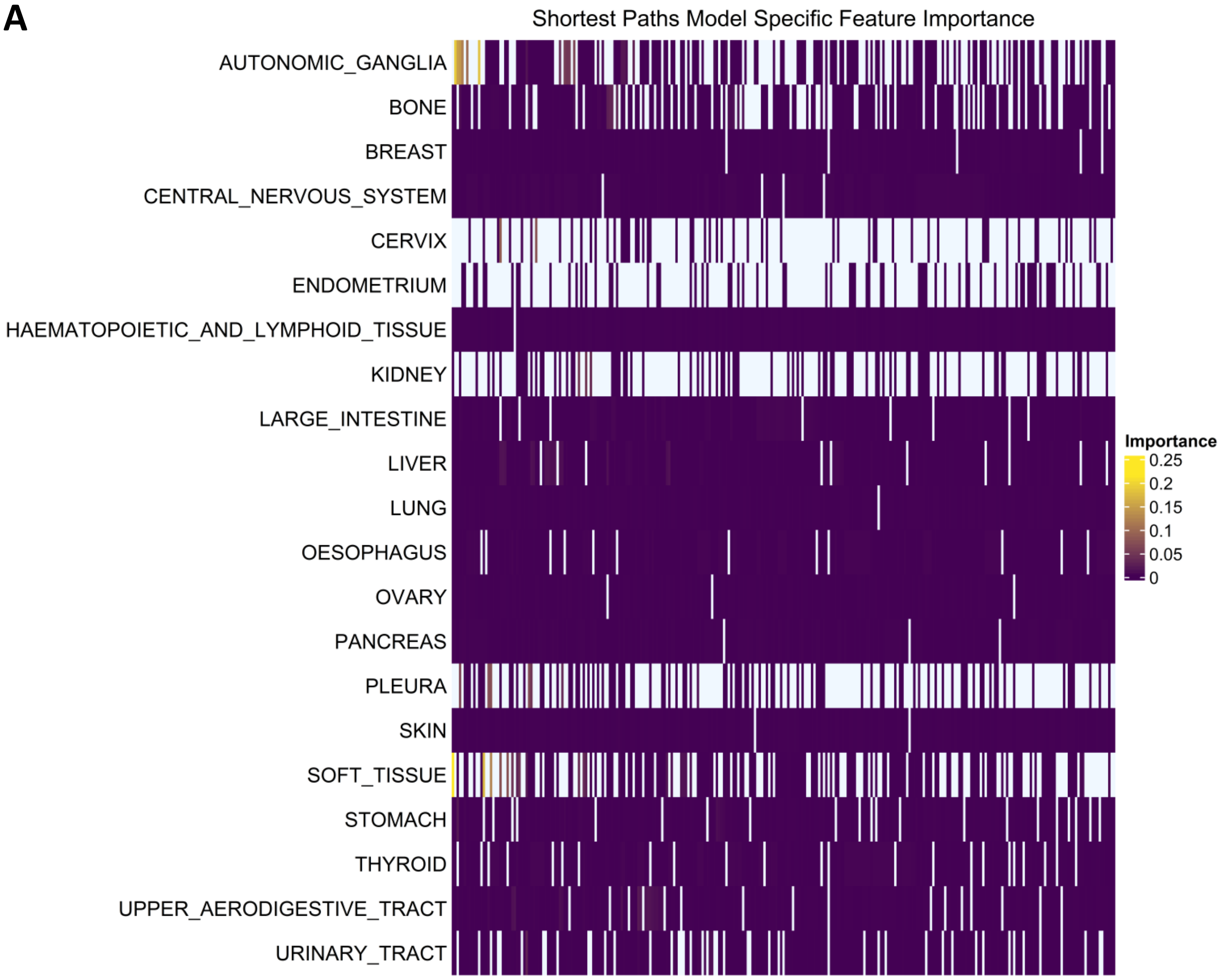

Shortest Paths Model Specific Feature Importance
AUTONOMIC_GANGLIA
BONE
BREAST
CENTRAL_NERVOUS_SYSTEM
CERVIX
ENDOMETRIUM
HAEMATOPOIETIC_AND_LYMPHOID_TISSUE
KIDNEY
LARGE_INTESTINE
LIVER
LUNG
OESOPHAGUS
OVARY
PANCREAS
PLEURA
SKIN
SOFT_TISSUE
STOMACH
THYROID
UPPER_AERODIGESTIVE_TRACT
URINARY_TRACT
Importance
0.25
0.2
0.15
0.1
0.05
0

Figure S5

A

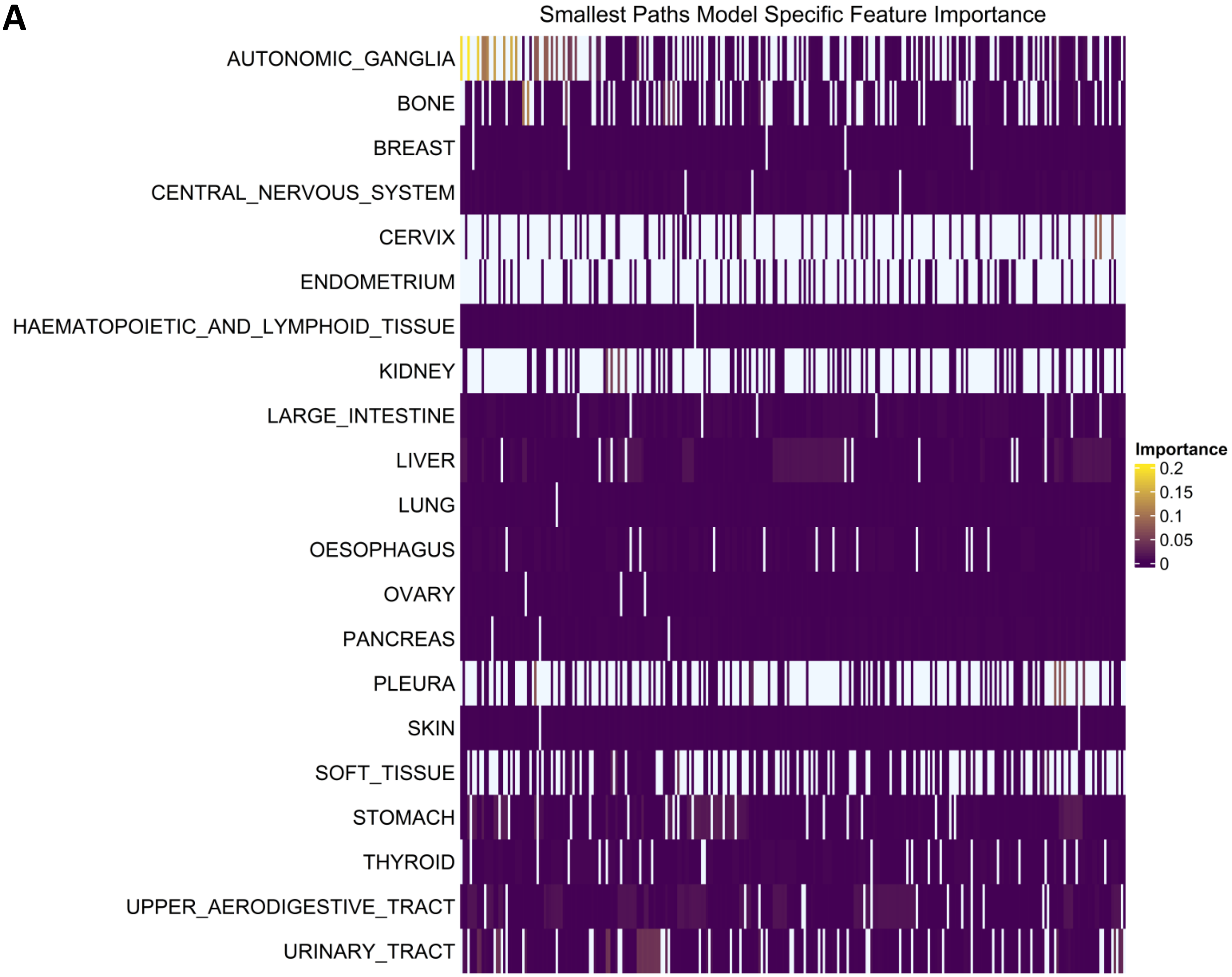
Smallest Paths Model Specific Feature Importance
AUTONOMIC_GANGLIA
BONE
BREAST
CENTRAL_NERVOUS_SYSTEM
CERVIX
ENDOMETRIUM
HAEMATOPOIETIC_AND_LYMPHOID_TISSUE
KIDNEY
LARGE_INTESTINE
LIVER
LUNG
OESOPHAGUS
OVARY
PANCREAS
PLEURA
SKIN
SOFT_TISSUE
STOMACH
THYROID
UPPER_AERODIGESTIVE_TRACT
URINARY_TRACT
Importance
0.2
0.15
0.1
0.05
0

Figure S6
